# Deep Learning Network for Speckle De-Noising in Severe Conditions

**DOI:** 10.3390/jimaging8060165

**Published:** 2022-06-09

**Authors:** Marie Tahon, Silvio Montrésor, Pascal Picart

**Affiliations:** 1 LIUM (Laboratoire d’Informatique de l’Université du Mans), Le Mans Université, Avenue Olivier Messiaen, 72085 Le Mans, France; marie.tahon@univ-lemans.fr; 2 LAUM (Laboratory of Acoustics of Le Mans Université), CNRS 6613, Institut d’Acoustique-Graduate School (IA-GS), Le Mans Université, Avenue Olivier Messiaen, 72085 Le Mans, France; pascal.picart@univ-lemans.fr; 3 ENSIM (École Nationale Supérieure d’Ingénieurs du Mans), Le Mans Université, Avenue Olivier Messiaen, 72085 Le Mans, France

**Keywords:** digital holography, image de-noising, deep learning, database controlled parameters, DnCNN, fine-tuning

## Abstract

Digital holography is well adapted to measure any modifications related to any objects. The method refers to digital holographic interferometry where the phase change between two states of the object is of interest. However, the phase images are corrupted by the speckle decorrelation noise. In this paper, we address the question of de-noising in holographic interferometry when phase data are polluted with speckle noise. We present a new database of phase fringe images for the evaluation of de-noising algorithms in digital holography. In this database, the simulated phase maps present characteristics such as the size of the speckle grains and the noise level of the fringes, which can be controlled by the generation process. Deep neural network architectures are trained with sets of phase maps having differentiated parameters according to the features. The performances of the new models are evaluated with a set of test fringe patterns whose characteristics are representative of severe conditions in terms of input SNR and speckle grain size. For this, four metrics are considered, which are the PSNR, the phase error, the perceived quality index and the peak-to-valley ratio. Results demonstrate that the models trained with phase maps with a diversity of noise characteristics lead to improving their efficiency, their robustness and their generality on phase maps with severe noise.

## 1. Introduction

Digital holographic interferometry is an indicated approach for contactless measurements at a large variety of scales [1]. The approach yields the difference in the light paths in a phase pattern which is wrapped modulo 2π. The phase generally refers to the “phase fringe pattern”, that is, an image exhibiting structured lines whose values are ranged in the [−π,+π] interval. This phase pattern is related to the inspected structure and permits us to address many industrial problems in connection with surface roughness, surface shape, surface deformation or surface vibration [2]. Digital holographic interferometry yields contactless, non-intrusive and full-field measurements. The non-intrusive feature is related to the use of coherent laser light to illuminate the object under interest. Basically, a speckle is issued from the structure which is illuminated by a laser, and is changed when the initial state of the object changes. As a consequence, the digital holograms include a speckle. It follows that when the speckle changes, phase noise is induced in the phase changes. Thus, de-noising the noisy phase is required and is carried out by considering filtering schemes [3]. The problem is then to de-noise the phase maps degraded with decorrelation phase noise. A broad discussion about the noise issue in digital holography is proposed in [4]. In [5], a recent review about the problem of image de-speckling in coherent metrology using deep learning based approaches is proposed. In [3] is demonstrated that the two dimensional windowed Fourier transform (WFT2F) is the best algorithm for de-noising in speckle-based metrology. The disadvantage of the WFT2F is the processing time required to de-noise phase data including 1024×1024 pixels. It follows that one requires fast de-noising processing for digital holographic interferometry. In order to achieve this goal, two directions must be considered. The first one is related to the modelling of the speckle decorrelation noise and will be not discussed in this paper. The second one is related to the use of artificial intelligence (AI) in order to increase the computation speed of the data processing. In [6,7], we proposed accurate modelling of the speckle noise. The advanced modelling of the speckle decorrelation noise permits us to carry on realistic simulations of phase data. Indeed, with natural images, there does exist a wide diversity which permits us to efficiently train AI with a large number of parameters. However, in the field of phase data processing in digital holography, the quantity and the diversity are strongly reduced. So far, it is not possible to obtain experimental phase data with speckle noise together with its clean version. That is the reason why realistic simulated data are required. Simulated phase data can thus be used for AI and deep learning based on convolutional neural network in order to address the processing of de-noising phase maps. Especially, the great interest in AI methods is that they may strongly decrease the processing time.

Related works on residual learning and holographic image databases are presented in Section 2. The aim is to reduce the de-noising time while reaching good performances on unseen images. To do so, new databases for training, development and validation are presented in Section 3. Residual learning architectures and their implementation are presented in Section 4. The baseline de-noising algorithms and protocols and metrics are summarized in Section 5. The results are discussed in Section 6.

## 2. Related Works

Several works on the removal of speckle noise in images with deep learning, and especially convolutional networks, were proposed in optical coherence tomography [8], hyperspectral imaging [9], or using multi-scale decompositions [10]. Conditional GANs [11] have also been experimented on. Deep networks, i.e., networks with a high number of layers, are able to capture highly complex and non-linear functions. These deep networks need to be trained with large amount of data to avoid over-fitting. The training stage has a high computational cost and the storage requires high memory capacities. Therefore investigating the suitable depth of the network is necessary [12]. When small quantities of data are available, one solution is to artificially increase the amount of training data [13]. In [14], the authors proposed to de-noise natural images with additive Gaussian noise using deep convolutional neural networks (DnCNN) by learning the residual noise instead of learning the de-noised image directly. Residual learning has the advantage of limiting the problem of vanishing gradients that can occur with very deep networks. Such work has served as a basis for many image processing networks such as disease detection in tomato leaves [15], and more specifically for speckle de-noising [16]. However, the cited works have used pre-trained networks that were learnt on natural images with additive Gaussian noise.

Due to the complexity of simulating realistic speckle noise on holographic images, most of the neural networks used in this field are pre-trained on natural images corrupted with additive Gaussian noise. In [3], HOLODEEP, a database including 25 fringe patterns divided into 5 patterns and 5 different signal-to-noise ratios was generated with a realistic noise simulator [17] to foster the diversity of phase fringe patterns. To the best of our knowledge, this database is the only available for training a de-noiser adapted to our context, but has the disadvantage of simulating soft conditions in terms of fringe diversity, signal-to-noise ratio and speckle grain size. In [18], a DnCNN was trained for speckle de-noising and reaches good performances compared to other de-noising advanced techniques on images recorded under soft conditions. A previous work [19] presented how light DnCNN (with small depth) are able to de-noise images from HOLODEEP with a nice compromise between computation cost and performance. The advantage of fine-tuning a DnCNN pre-trained on natural images on phase images corrupted with speckle noise was also investigated.

Considering the previous works in literature, the contributions of the present article are as follows: (i) the description of a new database designed to train and evaluate a residual network for speckle de-noising in severe conditions; (ii) the release of pytorch implementation which includes data loader and data augmentation; (iii) the evaluation of phase error performances obtained by several DnCNN networks trained on different subsets of images.

## 3. DB128: A Generalized Database for Digital Holography

### 3.1. HOLODEEP Database

In the previous paper [19], the authors presented a database, namely HOLODEEP, which includes five different types of noise-free phase fringe patterns. The database was considered for training the models and also for development. Each phase pattern of HOLODEEP was corrupted with realistic speckle noise having suitable statistics as described in [3]. The speckle grain size is fixed to $N_{s}=2$. Associated with the noise-free fringe patterns, the simulator [3] generated five noisy fringe patterns controlled with an adjustment parameter (namely Δ). The five data were corresponding to signal-to-noise-ratios (SNR) in the range [3 dB–12 dB]. A total of 25 images sized 1024×1024 pixels were generated. This database was designed for speckle de-noising in soft conditions, with a small speckle grain size (two pixels per grain) and moderate signal-to-noise ratios.

### 3.2. DB128: An Extended Database

#### 3.2.1. Definitions

In the present paper, networks are evaluated in terms of phase errors and generalization power defined as the “ability to perform well on previously unobserved inputs” [20]. In order to properly evaluate the generalization power of the network, the database is usually split in three subsets. The training subset consists of images used to train the network (∼80%). The development subset is used to verify if the network do not over-fit on the training data and is used to tune hyper-parameters (number of epochs, batch size, learning rate, etc.) (∼10%). Finally, the test subset is used to give the final performance of the trained network on unseen data (∼10%). This protocol ensures training and properly evaluating the neural networks: the images used to assess the model have not been seen by the network during the training stage.

#### 3.2.2. Diversity of Conditions

**Figure 1:**
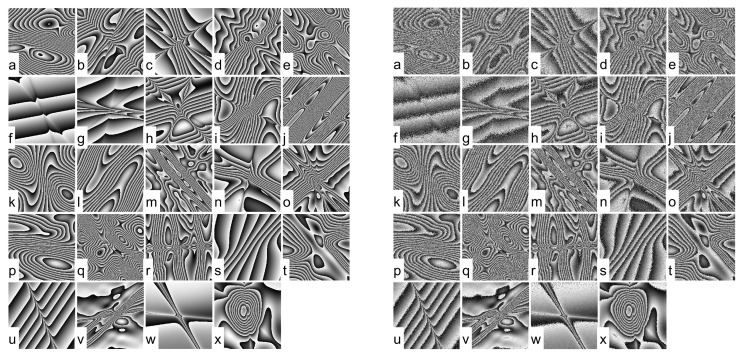
Phase maps of DB128-test. On the **left**, the 24 noise-free phase maps from (**a**–**x**), on the **right**, from (**a**–**x**) corresponding to 24 noisy phase maps with increasing input SNR.

Compared to [18], a new database, namely DB128 in the following, was generated using the realistic simulator. Phase fringe patterns with various signal-to-noise ratio (SNR), different speckle grain sizes and fringe diversity were simulated. DB128 includes 128 images of phase fringe patterns sized 1024×1024 pixels. Input cosine SNRs in DB128 are in the range [−0.2, 8] dB. Three different speckle grain sizes have been used to generate images, at $N_{s}=\left(2,4,6\right)$ pixels per grain. In order to train and test the deep learning model, DB128 has been split in three sets: a training database DB128-train with 94 images, a development database with 10 images and a testing database, named DB128-test with 24 images. Images of DB128-test devoted to the evaluation of the models are reported in Figure 1. Note that a specific parameter Δ is used in the simulation to increase the noise amount in order to simulate severe experimental conditions [3,18]. More precisely, database DB128 includes images that were generated to have a low input signal-to-noise-ratio (less than 3 dB) and a speckle grain size up to six pixels per grain. The notion of “severe conditions” corresponds to the joint realization of these conditions that are found in a lot of phase maps of this database.

DB128 generation was driven to cover a high diversity of interference fringes. To preserve this diversity, we decided to keep Ns and Δ distributions in all subsets as shown in Table 1. In the end, 94 images were used to train the networks, while 24 images were used to evaluate the performances. Hyper-parameters and network optimizations have been carried out in previous work (see [19]). Consequently, the development set is not used in the present experiments.

## 4. Residual Learning Architectures

### 4.1. Architectures

DnCNN is a residual network which originally includes 59 layers. DnCNN is organized according to a first input layer (3×3 convolutional layer and rectified linear units ReLU), 16 intermediate convolutional blocks (ConvBlocks: 3×3×64 convolutional layer, batch normalization and ReLU), and one output layer (3×3×64 convolutional layer). The latter layer permits us to reconstruct the output noise. The de-noised image is obtained by calculating the difference between the input noisy image and the output noise. The L2 loss between the reference and the predicted pixel values is considered for the loss function. For the reader interested in the architecture, please refer to the DnCNN original model (https://github.com/wbhu/DnCNN-tensorflow (accessed on 14 April 2022)).

In a previous work, two different depths for DnCNN have been investigated [19]. We have shown that DnCNN with only four ConvBlocks can be adequate when training HOLODEEP benchmark database. The advantage of such light networks is that they are trained quickly and they do not require large memory during inference. However HOLODEEP is not a highly diverse database in comparison to DB128, and we are not sure that light networks will be able to de-noise phase maps with high diversity.

### 4.2. Implementation

The implementation with Tensorflow was first considered and then adapted using the PyTorch framework (https://git-lium.univ-lemans.fr/tahon/dncnn-tensorflow-holography/ (accessed on 14 April 2022)).

In the original version, two main scripts were used: one for the patch generation, and one for the training and evaluation of the models. In order to foster the computation time, a dataloader is implemented instead of the patch generation script. The dataloader automatically extracts from original complete images a given number of patches, shuffles them, converts radian phases to cosine or sine images, and performs artificial data augmentation.

Artificial data augmentation aims at creating more training data by the use of standard image transformations. This operation is performed directly on patches, and generally avoids over-fitting. Possible patches transformations are as follows:

- *add45*: add π/4 to phase;
- *transpose*: transpose phase;
- *flip*: flip up down cosine/sine images;
- *rot90*: π/2 rotation of cosine/sine images;
- *rot180*: π rotation of cosine/sine images;
- *rot270*: 3π/2 rotation of cosine/sine images.

These transformations can apply directly to the phase image or to its cosine or sine version. Most of them are cumulative. For example, data augmentation *add45transpose* aims at creating four times more patches as the transposition applies to the original phase and the phase +π/4. The network depth is controlled with parameters *D* which corresponds to the number of ConvBlocks in the DnCnn architecture, and *C* which is the convolution kernel size. All the training parameters are set up in yaml file: patch size, batch size, learning rate and the maximum number of epochs.

## 5. Algorithms and Metrics

### 5.1. Challenger Algorithms

In order to compare the deep learning approach written in Pytorch, several challenger de-noising algorithms were chosen from literature. So we did select four algorithms that previously demonstrated to be efficient in our context. These are: The block-matching 3D filter [21] (BM3D), the windowed Fourier transform [22] (WFT2F), the dual-tree discrete wavelet transform (DTDWT), which is a particular implementation of the dual-tree complex wavelet transform [23] and finally what we call DLSHIFT algorithm, which was our last deep learning based approach write in Matlab presented in a recent paper [5]. The block-matching 3D (BM3D) algorithm takes into account both local and non-local features of data and is built on the concept of grouping and collaborative filtering. Grouping finds and stack together in dedicated arrays mutually similar image blocks. Collaborative filtering provides individual estimates of all grouped blocks jointly. The BM3D algorithm is considered the best de-noising algorithm for natural images. WFT2F computes a local Fourier transform and thus can take into account of noise non-stationary feature. The WFT2F algorithm is specially adapted for phase de-noising in speckle metrology since its input is a complex phase generated by the complex-valued exponential of the initial phase image. The DTDWT is another substitute to the stationary discrete wavelet transform because it yields significant enhancements for image de-noising. DTDWT is nearly shift invariant and direction selective in the 2D domain. This property is obtained considering a redundancy factor of only 2D for d-dimensional signals which is substantially lower than the stationary discrete wavelet transform usually involved in image processing for de-noising applications. The DTDWT is built with a set of separable filters which make the algorithm computationally very efficient. DLSHIFT is residual deep learning based approach written in Matlab. The algorithm is based on a DnCnn model [14] which has been firstly trained with a database consisting of natural images. It then has been retrained with a second database of phase images with decorrelation noise using the simulator. The DLSHIFT algorithm combines the use of several iterations with a phase shifted procedure in order to get multiple realisations of a unique input phase map. This scheme allows performing as much as the WFT2F does with the the HOLODEEP database. In order to evaluate the efficiency of iterations of our Pythorch model, we compare three versions which are called DL_DB128, DL_DB128_2 and DL_DB128_3. The two latter correspond to the cascade of respectively two and three DL_DB128 procedure.

### 5.2. Metrics

Quality metrics are needed in order to appraise the performances of the algorithms. As the application field of this work is speckle metrology, the criterion based on the phase error is particularly adapted. The phase error is obtained by calculating the difference between the reference noise-free phase and the de-noised one. Then, the standard deviation of the residue is calculated. The phase error has intrinsically a downside because based on the calculation of an average phase error over the set of pixels. It follows that it does not account for distortions which could disturb the structures in the phase data. Consequently, a similar error can be seen between two de-noised phase data whereas one can have a completely distinctive perceived quality. For this reason, the “quality index”, Qindex, criterion from Wang and Bovik [24] was designed to evaluate the perceived quality of processed images. The Qindex criterion reaches the value of 1 when the two images are completely identical. In this work, the quality index is considered for verifying the perceived quality. Note also that the peak signal-to-noise ratio (PSNR) is the most used criterion in papers dealing about image restoration. The PSNR is computed in dB from the ratio of the squared maximum level in the image with the mean squared error between the reference image and the processed ones. In a previous paper [25], it has been shown an empirical numerical link between the PSNR and the phase error. Finally, the last criterion that is often used in digital holography interferometry is the peak-to-valley (PV) signal to noise ratio. It expresses the absolute difference value of the minimum and the maximum computed from the residue error. The PV is commonly, but not only, used in papers that deal with phase unwrapping problems [26]. The reader interested by the topic may find the formal definitions of the metrics used in this evaluation in previous papers from the authors [3,18,25,26].

## 6. Results

### 6.1. Protocol

We have trained diverse DnCNN networks in order to investigate the impact of the diversity of the training database, the network depth (*D* parameter, i.e., the number of ConvBlocks), the convolutional kernel size (*C*). To compare with our previous work, we trained DnCNN models with HOLODEEP images as our baseline model with D=4; 16 ConvBlocks. In order to investigate how the diversity of the training data impacts model performances, we also trained DnCNN models with the entire DB128 (DB128-full) and the train set described in Table 1. The kernel size has also been studied (C=16;32;64). However, a kernel sized 64 pixels produces significantly better results than lower sizes. Therefore, this parameter is set to 64 in the following.

Training images sized 1024×1024 are cropped into 384 patches of size 50×50, randomly selected among all possible patches, and data augmentation is performed with *add45transpose*, thus augmenting four times the number of training patches. The learning rate is 0.001 and the batch size is 64 patches per iteration. The loss function is standard RMSE between predicted and reference patches. The maximum number of epochs is set to 100 in order to have the same number of iterations for each model.

### 6.2. Global Evaluation

**Figure 2:**
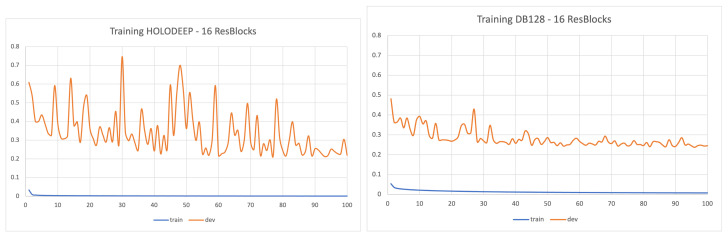
Evolution of the loss function (RMSE) at each epoch when training on HOLODEEP (**left**) or DB128 (**right**). Mean obtained on the training dataset (blue) and the development set, here are the complete images from HOLODEEP (orange).

The trained models are evaluated on the basis of the average phase error (Δϕ in rad) on the test images as detailed in Table 2. In order to estimate the generalisation power of the models on unseen complete images sized 1024×1024, three test subsets are used: HOLODEEP (25 images), DB128-full (128 images) and DB128-test (24 images). Figure 2 shows the evolution of the loss function (RMSE) during the training stage evaluated on both the training patches and complete images from HOLODEEP (our development subset) for two networks trained on HOLODDEP and the full DB128 subsets. The epoch corresponding to the lowest loss is always the last epoch 100.

Table 2 shows that deeper models (D=16) reach better performances either on seen or unseen data than light models (D=4). However, light models can be relevant for real-time applications as they speed-up the de-noising process. Indeed, inference is almost four times faster with D=4 (80 ms on a RTX600 GPU card) than D=16 (310 ms).

First, regarding the performances obtained on HOLODEEP (3rd column), it is evident that training and evaluating a model on the same database aims at the best results (Δϕ=0.0391 rad). However, the model trained on DB128-train achieves very competitive results (Δϕ=0.0437 rad) beside the fact that it is not trained on the same data used for the evaluation. Second, the performances obtained on DB128-full and DB128-test (columns 4 and 5) are almost similar. It shows that the way the full database is split along the two controlling parameters Δ and $N_{s}$ conserves the diversity of the images. Finally, when evaluating the DB128-test, the difference between models trained on DB128-full (Δϕ=0.1320 rad) and DB128-train (Δϕ=0.1290 rad) is very low. The results show that the model trained on DB128-train achieves a better performance than the model trained with the full database on both the same full database and the test subset. During the training stage, the models are optimized on patches of size 50×50, while during the evaluation, the full 1024×1024 images are evaluated. This mismatch could explain the differences between these two models. Nevertheless, the last model trained on 94 images has the advantage of being trained on different data.

All these conclusions show the high generalization power of the network trained on DB128-train on both HOLODEEP and DB128-test evaluation databases. It confirms the importance of a controlled diversity in the training images. A closer look to Figure 2 shows that loss calculated on the development set is much more variable across epochs when the model is trained on HOLODEEP than on the full DB128 database. This confirms the high generalization power and robustness to unseen data of a de-noiser network trained on DB128.

Applying the de-noising process many times on a noisy image is also possible. The results of three successive process show that it is still possible to gain in performance up to 2 iterations: Δϕ=0.1290 rad (1 iteration), Δϕ=0.1194 rad (2 iterations) and Δϕ=0.1297 rad (3 iterations).

**Figure 3:**
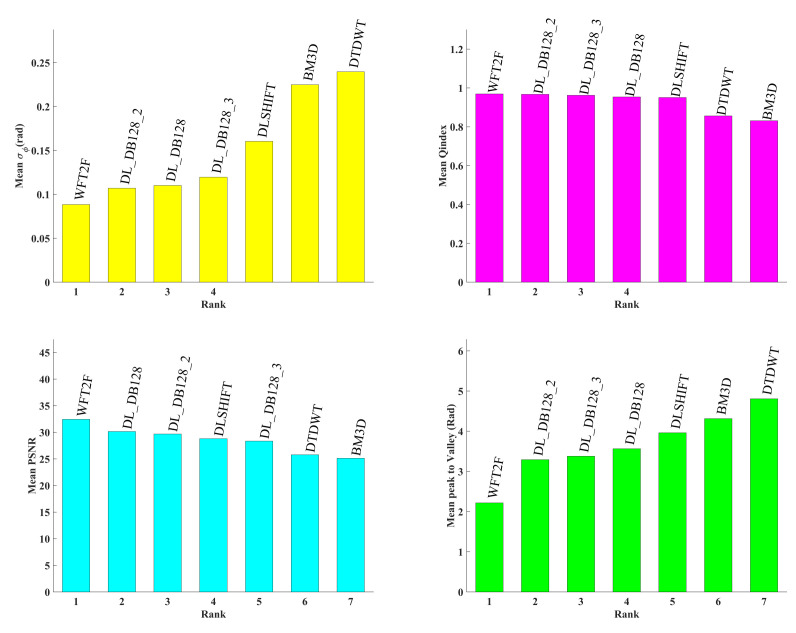
Rankings of metrics on DB128-test. From **left** to **right** and **top** to **bottom**: phase error, Qindex, PSNR and Peak-to-valley.

**Figure 4:**
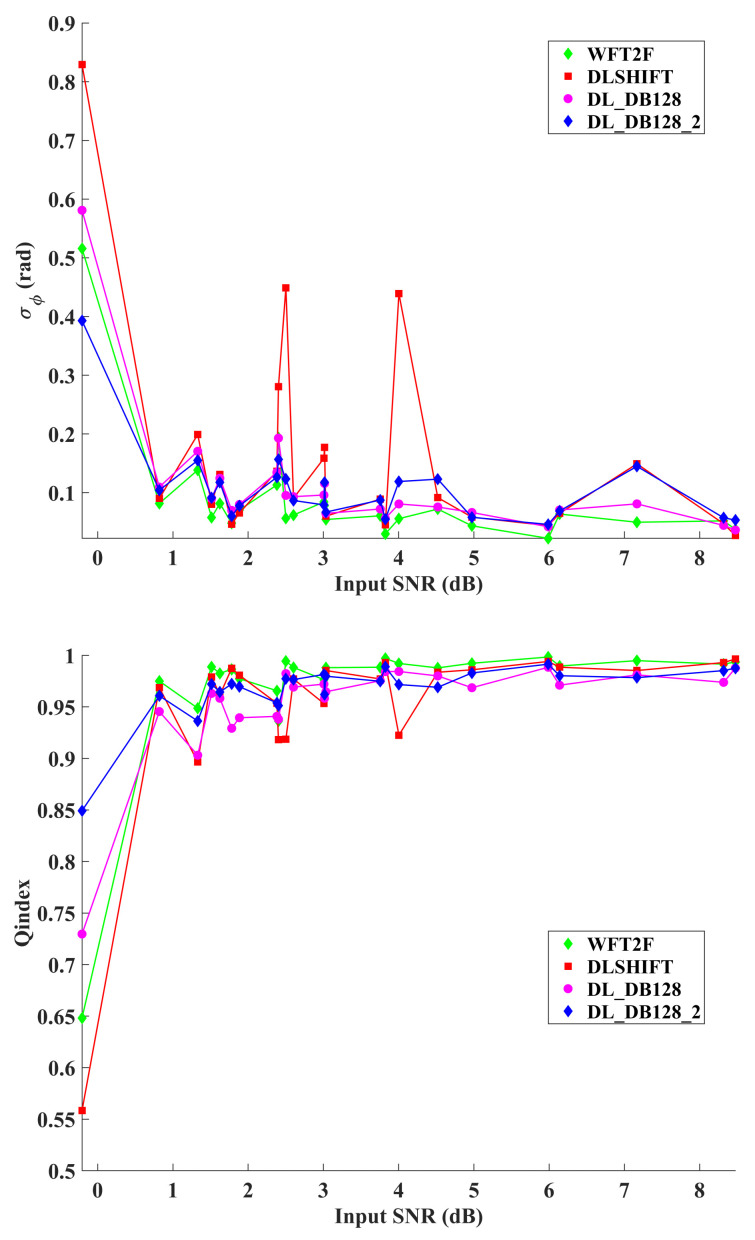
Trends of metrics vs input cosine SNR for 4 selected algorithms. From **top** to **bottom**: phase error, Qindex and PSNR.

Assessments of previous presented algorithms are reported in Figure 3 and Figure 4. Figure 3 provides rankings of the seven algorithms for the four selected metrics evaluated on DB128-test database. It appears that WFT2F remains the best algorithms with a mean phase error of about 0.09 rad. Previous evaluations have shown that this method achieved 0.025 rad with the HOLODEEP database which was constituted only with phase maps degraded with speckle noise with two pixels per grain. The residual deep learning model DL_DB128 provides very close results with WFT2F. Particularly, the use of two iterations successively yields a phase error of 0.12 rad. Then, DLSHIFT algorithm achieved no better phase error than 0.16 rad due to the fact that this model had been trained with speckle grain of size 2. Finally, BM3D and DTDWT demonstrate that they strongly fail in that context. Similarly, the same comments can be made about ranking of Qindex and peak- to-valley metrics. Trends of metrics are reported in Figure 4. Only the four best algorithms of the phase error ranking are reported. One can clearly see the consequence of using iterations with the deep learning model. For low input SNR below 3dB, DL_DB128_2 performs systematically better than DL_DB128. Beyond 3 dB, the contrary is observed. Note also that DLSHIFT under performs other algorithms with cases corresponding to speckle size larger than two pixels per grain, respectively for −0.2 dB, 2.5 dB and 4.1 dB of input SNR. The reason is that the model was trained only with phase maps degraded with speckle noise with size 2. Equivalent comments can be made with the Qindex metric.

### 6.3. Evaluation on Target Images

**Figure 5:**
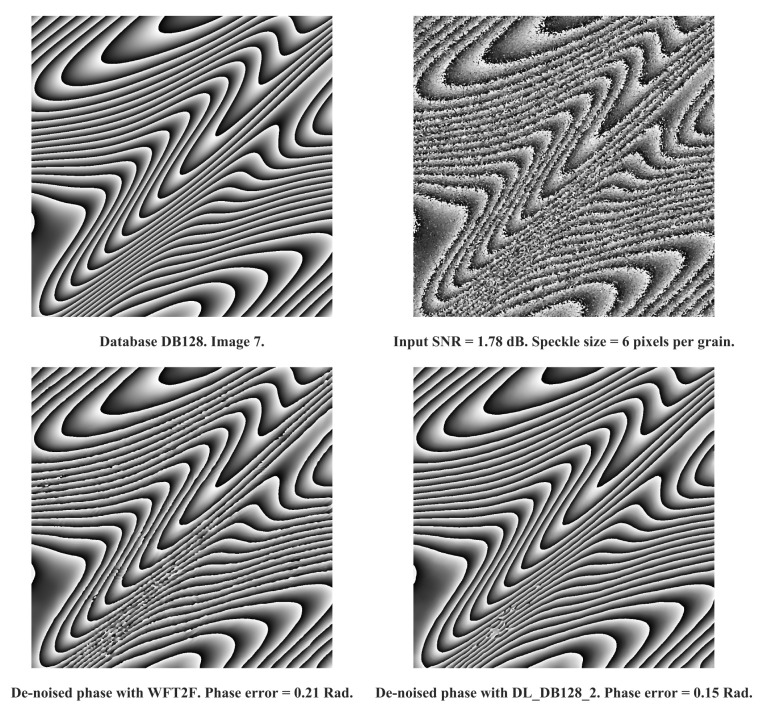
Comparison between WFT2F and DL_DB128_2 with image 7 of DB128-train database. From **left** to **right** and **top** to **bottom**: noise-free phase, noisy input phase, de-noised phase with WFT2F and DL_DB128_2. Cosine input SNR is −0.2 dB. Phase error with WFT2F is 0.21 rad. Phase error with DL_DB128_2 is 0.15 rad.

**Figure 6:**
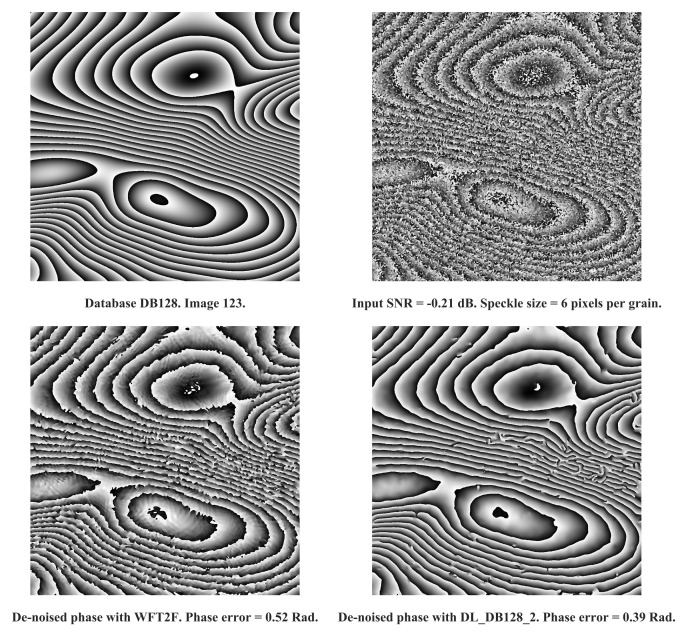
Comparison between WFT2F and DL_DB128_2 on image 123 of DB128-test database. From **left** to **right** and **top** to **bottom**: Noise-free phase, noisy input phase, de-noised phase with WFT2F and DL_DB128_2. Cosine input SNR is of 1.78dB. Phase error with WFT2F is equal to 0.52 rad. Phase error with DL_DB128_2 is 0.39 rad.

In Figure 5 and Figure 6 are reported examples of processing achieved with DL_DB128_2 and WFT2F. Examples show bad conditions of degradation from input SNR and speckle size characteristics. For both examples, we clearly assess the efficiency of deep learning based model with respectively a difference of phase error of 0.06 rad and 0.12 rad for image 7 and image 123 of DB128 database. That demonstrates that DL_DB128_2 appears to be the best algorithm to deal with severe conditions.

## 7. Conclusions

This paper focuses on the problem of the de-noising of holographic phase images and presents a deep learning based approach that is specific for strong speckle noise. We introduce a new database named DB128 to train a residual model so that it better processes diverse and severe noise conditions. Particularly, the severe conditions deal with very low input SNR below 0dB, and large speckle grains, typically more than four pixels per grain. The new database constituted with 128 phase fringe patterns sized 1024×1024 pixels is characterized by a large diversity of its features: fringe density, fringe orientation, input SNR, speckle size, and fringe curvature. Results demonstrate that the model trained with the new database outperforms the two-dimensional windowed Fourier transform method with phase maps which correspond to severe conditions. Moreover, this model better generalizes to unseen phase maps than another model trained with the former HOLODEEP database. Further works intend to improve speckle de-noising by combining the advantages of the two approaches by using local estimators of the fringe density, input SNR and speckle grain in the input phase map in order to obtain efficient mixing of both approaches.

## Figures and Tables

**Table 1:** Distribution of parameters within phase images.

Parameters	Total	Train	Dev	Test
Ns=2	57	42	5	7
Ns=4	33	28	3	5
Ns=6	38	24	2	4
0≤Δ<2	58	42	4	7
2≤Δ≤4	70	39	4	7
Total	128	94	10	24

**Table 2:** Δϕ (rad) obtained by DnCNN models with 4 or 16 ConvBlocks trained on HOLODEEP, or DB128. The averaged Δϕ (rad) is given on our three test databases (in average).

Training Data (Nb of Images)	Depth *D*		Test Data DB128-Full	
		HOLODEEP		DB128-Test
HOLODEEP (25)	4	0.0583	0.2816	0.2858
HOLODEEP (25)	16	0.0391	0.2505	0.2623
DB128-full (128)	4	0.0650	0.2341	0.2291
DB128-full (128)	16	0.0477	0.1331	0.1320
DB128-train (94)	4	0.0657	0.2394	0.2467
DB128-train (94)	16	0.0437	0.1240	0.1290

## Data Availability

HOLODEEP database is freely available [DOI:10.13140/RG.2.2.20819.78885 (accessed on 14 April 2022)].
